# Impact of a stigma-disrupting addiction medicine core curriculum on internal medicine resident attitudes and preparedness: results from a pilot study

**DOI:** 10.1186/s12909-026-10119-8

**Published:** 2026-08-12

**Authors:** David Lawrence, Katharine F. Marshall

**Affiliations:** 1 VA Greater Los Angeles Health System, Department of Mental Health, UCLA David Geffen School of Medicine, Department of Medicine, Los Angeles, CA USA; 2 Department of Addiction Medicine, Kaiser Permanente Northwest Medical Group, Portland, OR USA

**Keywords:** Graduate medical education, Substance use disorder, Curriculum development, Addiction medicine, Stigma

## Abstract

**Background:**

Amidst the worsening United States drug overdose crisis, the Accreditation Council for Graduate Medical Education (ACGME) instituted requirements for internal medicine (IM) residency programs to provide trainees with core substance use disorder (SUD) instruction and clinical experiences. Negative provider attitudes towards patients with SUDs, however, are a significant barrier to learning and providing standard-of-care treatment. We designed an addiction medicine curriculum for IM residents combining transformative learning methods and core competencies in SUD care with stigma-disrupting learning objectives and messaging strategies. The curriculum was implemented as a five-day core rotation for second year IM residents at a large, urban academic program. Here we report the results of a pilot study evaluating the curriculum’s association with self-reported attitudes towards and preparedness to diagnose and treat patients with SUDs.

**Methods:**

All participating residents received voluntary pre- and post-experience surveys evaluating self-reported preparedness to diagnose and treat SUDs on a 4-point Likert scale and attitudes towards patients with opioid and alcohol use disorder on the Medical Condition Regard Scale (MCRS). Responses were linked by anonymous unique identifier and analyzed with McNemar’s and paired t-test. The evaluation was approved by the program’s Institutional Review Board.

**Results:**

Of residents completing the curriculum, 18 of 46 (39%) also completed both the pre- and post- survey. Of those, preparedness to diagnose SUDs increased over the course of the curriculum from 39% to 100% (*p* < 0.05) and preparedness to treat increased from 22% to 89% (*p* < 0.05). Average MCRS scores also demonstrated significant increases for both opioid (41.9 to 47.2, *p* = 0.003) and alcohol use disorders (41.3 to 47.1, *p* < 0.001), indicating increased regard for patients with these conditions at curriculum completion.

**Conclusions:**

Our findings build on previous work associating stigma-reducing educational strategies with increased preparedness to diagnose and treat SUDs conditions in a non-self-selected core rotation learner cohort. Medical educators should consider incorporating these strategies when developing ACGME-mandated addiction medicine curricula for IM residents.

**Supplementary Information:**

The online version contains supplementary material available at https://doi.org/10.1186/s12909-026-10119-8.

## Background

Drug overdose is now the leading cause of death in U.S. adults under 55, with more than 15% of American adults currently meeting criteria for at least one substance use disorder (SUD) [1, 2]. Despite the high mortality and prevalence of disease, fewer than 10% of people with SUD report receiving treatment for it in the last year [1]. One critical barrier to treatment access is the low number of generalist providers who are willing and able to provide it. A 2016 survey showed that fewer than 10% of internists felt “very prepared” to discuss evidence-based medication or behavioral treatments with their patients with SUD; a 2017 survey reported that only 20% of primary care providers had interest in treating patients with opioid use disorder [3, 4]. Accordingly, the Accreditation Council for Graduate Medical Education (ACGME) released updated requirements in 2022 for internal medicine residency programs to implement core SUD instruction and clinical experiences for all trainees [5].

But effective implementation of the ACGME requirement must take into account why this training gap exists in the first place. Lack of interest in treating and lack of preparedness are reflections of stigma, manifested as unequal treatment, discrediting thoughts and actions, and a desire for social distance from the stigmatized group [6]. The existence and application of stigma against patients with SUD by healthcare providers and systems are well established [7]. Degree of provider stigma, sometimes also described as “negative attitudes” or “low regard,” is directly correlated with failure to provide or refer for appropriate treatment for patients with SUD [3, 8].

Physicians’ already negative attitudes and low interest in treating patients with stigmatized conditions including SUDs, schizophrenia, and depression deteriorate over the course of residency; among these, the largest decrease in regard is seen for patients with SUDs [9, 10]. Thus, graduate medical education is a useful setting for an intervention designed to counteract these attitudinal changes, reduce stigma, and promote interest in treating patients with SUD.

Prior curricular interventions in this area have sought to improve preparedness to provide SUD care or reduce stigma, but rarely both [9, 11, 12]. One study delivered a 10-hour curriculum to internal medicine residents and measured resident regard before and after in a first-year cohort but found no significant change in regard after the curriculum [13]. Another delivered 5 hours of content on SUD to first-year surgery and medicine residents and found increased knowledge scores but no change in attitudes [14].

We designed, implemented and evaluated a comprehensive curriculum in SUD care for internal medicine residents using anti-stigma learning objectives and messaging techniques with the aim of concurrently increasing resident practice preparedness as well as positive regard for patients with SUDs. 

## Methods

### Setting and participants

All second-year categorical internal medicine residents at an urban academic hospital were scheduled to participate in the 1-week core rotation. The rotation is composed of educational experiences (see program description for detail) and hands-on clinical experiences comprised of 2 full days at an outpatient specialty addiction treatment clinic offering psychosocial treatment and office-based pharmacotherapy for SUDs and methadone for opioid use disorder through a co-located Opioid Treatment Program, and 3 full days on an inpatient addiction medicine consult service at an urban academic medical center. While case-mix and trainee exposure varies from week to week, these settings routinely care for patients with a high prevalence of alcohol, opioid, and stimulant use disorders across varying stages of severity and stability.

### Program description

Core competencies in SUD care for internal medicine residents were identified by Jackson and colleagues in 2010, with attitude change noted as a key strategy for educational change (Appendix A) [15]. We augmented these competencies to also include learning objectives specifically focused on stigma: the impact of stigma on patients with SUDs, principles of trauma-informed care in the management of patients with SUDS, key principles and strategies of harm reduction, interactive training in core motivational interviewing skills, and an end-rotation reflection session.

Content was divided across five pre-recorded video didactic lectures presented by one of the authors (KM) (fundamentals, policy issues, opioid use disorder, alcohol use disorder, stimulant and sedative use disorders), and three attending-facilitated interactive sessions (screening brief intervention and motivational interviewing skills, ethical issues in SUDs, end-rotation reflection). Table 1 summarizes the curriculum, and Appendix B lists the detailed learning objectives.

**Table 1:** Curriculum overview

Session Title	Format	Core Competencies (Jackson et al. 2010) [15]*
Fundamentals of Substance Use Disorders Care	Asynchronous video	1, 6
Screening, Diagnosis, Brief Intervention and Motivational Interviewing	Facilitated workshop	1, 2, 3, 4, 5
Policy Issues in Substance Use Disorders	Asynchronous video	2, 5
Ethical issues in Substance Use Disorder Care	Facilitated workshop	8, 9
Opioid Use Disorder	Asynchronous video	5, 6, 7, 10, 11
Alcohol Use Disorder	Asynchronous video	5, 6, 7, 10, 11
Stimulant and Sedative Hypnotic Use Disorder	Asynchronous video	6, 7, 10, 11
Reflections in Care of SUD	Facilitated discussion	

*Key to recommended competencies according to Jackson, et al., in brief: (1) Screening (2) Assessment (3) Brief intervention (4) Counseling (5) Referral for or provision of pharmacotherapy (6) Comorbid conditions (7) Referral for treatment and supportive services (8) Ethics and legal issues in physician impairment (9) Ethics and legal issues in patients with SUD (10) Withdrawal management (11) Relapse prevention

Four principles of transformative learning theory (disorienting dilemma, critical reflection, discourse, action) were incorporated throughout the curriculum [16]. Didactic sessions began with a case presenting learners with a ‘disorienting dilemma’ in the care of people with SUDs, followed by the delivery of learning objectives. Prior to the end of the rotation, residents were asked to write personal essays on their encounters with patients with SUDs (critical reflection) and during the end-rotation reflection session residents participated in a facilitated discussion with prompts that evoked reflection on the impact of stigma in the practice of medicine and potential changes for future practice (critical reflection, discourse, action) [16].

In addition to learning objectives and interactive sessions specifically focused on stigma, we incorporated four previously described stigma-disrupting messaging techniques to deliver all didactic session content [17]. First, we used person-centered language (i.e. “person with a SUD” instead of “addict”) consistently, and provided reminders about the importance of respectful language at the beginning of each session. Second, we incorporated sympathetic narratives about patients with SUD into each session using case-based learning. Third, we emphasized societal influences on SUD care including the role of trauma and heredity in risk for developing SUD and information about the role of social and racial discrimination in current substance use policy and treatment. Fourth, we emphasized treatability including data on the high efficacy of many treatments and positive outcomes in the cases presented. 

### Evaluation

The survey instrument was developed after a thorough review of the literature, with input from experts in the fields of addiction medicine and survey research. The pre-rotation survey solicited prior addiction medicine training experiences, and both pre- and post-rotation surveys solicited perceived preparedness to diagnose and preparedness to treat “substance use disorders” using 4-point Likert scale questions and stigmatized attitudes using the Medical Condition Regard Scale (MCRS), specifically for patients with “alcohol use disorder” (AUD) and “opioid use disorder” (OUD). Complete text of the survey instrument is available in Appendix C. The MCRS is a valid and reliable 11-item instrument assessment tool for healthcare professionals graded on a 6-point Likert scale, with higher scores indicating a higher perception of enjoyment, treatability, and belief in the utility of dedicating medical resources for a given condition; used as a proxy for attitude [18] that has been used in previous evaluations of stigma towards mental health and SUD diagnoses amongst post-graduate medical trainees [9, 13, 19, 20]. All resident rotators were emailed a personalized link to an online survey prior to the start of the rotation and at the end of the last day of the rotation. Residents were informed that participation was voluntary and that completion of the survey would indicate informed consent. This study received IRB approval from the University of California Los Angeles (Protocol IRB-22-0724) and complied with the Declaration of Helsinki, and residents provided consent to participate. 

### Statistical analysis

We collapsed the “very prepared” and “somewhat prepared” categories and the “very unprepared” and “somewhat unprepared” categories of the 4-point Likert scale survey questions evaluating preparedness and compared these using McNemar’s test. For MCRS results, means and standard deviations were calculated and compared using paired t-tests. Study data were collected and managed using Qualtrics, a secure web-based software platform hosted by the academic medical center. Data analysis was performed using IBM SPSS Version 28 January 2022.

## Results

Of the 48 residents scheduled to attend, 46 completed the rotation. Of those completing the rotation, 18 (39%) completed both the pre- and post-survey. Of those 18 completing both surveys, nine (50%) reported having no prior training in SUD care in medical school. The proportion of residents reporting being somewhat or very prepared to diagnose SUDs increased from 39% before to 100% after the curriculum (*p* < 0.05), and the proportion reporting feeling somewhat or very prepared to treat SUDs increased from 22% to 89%, (*p* < 0.05). Mean summative MCRS scores similarly had a significant increase for patients with OUD from pre (mean = 41.94, SD = 8) to post (mean = 47.2, SD = 7.5, *p* = 0.003) and for patients with AUD from pre (mean = 41.3, SD = 7.1) to post (mean = 47.1, SD = 7.2, *p* < 0.001) rotation (Table 2).

**Table 2:** Pre- and post-curriculum medical condition regard scale (*n* = 18)

	Pre	Post		Pre	Post	
Medical Condition Regard Scale	Opioid Use Disorder Mean (SD)	Opioid Use Disorder Mean (SD)	Pre-post Δ, *p* value*	Alcohol Use Disorder Mean (SD)	Alcohol Use Disorder Mean (SD)	Pre-post Δ, *p* value*
Working with patients like this is satisfying.	4.28 (1.1)	4.83 (0.9)		4.00 (0.9)	4.78 (1.0)	
Insurance plans should cover patients like this to the same degree they cover patients with other conditions.	5.44 (0.9)	5.67 (0.5)		5.11 (0.8)	5.56 (0.8)	
There is little I can do to help patients like this. ^R^	3.28 (0.9)	4.00 (0.8)		3.33 (0.9)	3.94 (0.8)	
I feel especially compassionate toward patients like this.	4.28 (0.8)	4.61 (0.9)		4.17 (1.0)	4.39 (1.1)	
Patients like this irritate me. ^R^	3.33 (1.1)	3.61 (1.1)		3.22 (1.1)	3.94 (0.8)	
I would not mind getting up on call nights to care for patients like this.	3.50 (1.0)	3.72 (1.4)		3.61 (1.3)	3.72 (1.4)	
Treating patients like this is a waste of medical dollars. ^R^	4.39 (0.7)	4.67 (0.6)		4.33 (0.6)	4.67 (0.6)	
Patients like this are particularly difficult for me to work with. ^R^	2.83 (1.3)	3.22 (1.2)		2.72 (1.0)	3.17 (1.2)	
I can usually find something that helps patients like this feel better.	3.11 (0.9)	4.44 (0.8)		3.56 (0.9)	4.67 (0.8)	
I enjoy giving extra time to patients like this.	4.11(1.0)	4.44 (1.1)		3.83 (1.0)	4.33 (1.1)	
I prefer not to work with patients like this. ^R^	3.39 (1.1)	3.94 (1.1)		3.39 (1.0)	3.89 (1.1)	
Mean Overall Likert Score	3.81 (0.7)	4.29 (0.7)	0.48, 0.003	3.75 (0.6)	4.28 (0.7)	0.53, < 0.001
Mean Overall Summative Score	41.94 (8.0)	47.17 (7.5)	5.23, 0.003	41.28 (7.1)	47.06 (7.2)	5.8, < 0.001

Scale: 1 = Strongly disagree; 2 = Disagree; 3 = Not sure but probably disagree; 4 = Not sure but probably agree; 5 = Agree; 6 = Strongly agree

^R^ Item is reverse scored (e.g. “strongly agree” = 1 instead of 6)

*p value from paired samples t-test (two-sided)

## Discussion

A one-week core clinical and educational experience including a stigma-disrupting core curriculum in addiction medicine for second year internal medicine residents was associated with significantly increased self-reported preparedness to diagnose and treat SUDs and improved positive regard for patients with opioid and alcohol use disorders.

Strengths of this pilot study include implementation as a core rotation, such that all second-year IM residents, instead of only a self-selecting cohort of elective residents, participated – a factor reducing the risk for selection bias in our results. Additional strengths include the size of the program, such that a relatively large number of trainees were exposed to the curriculum during the single year of evaluation. The versatility of the curriculum is another strength. While delivered as a 5-day core rotation, the curriculum can be easily adapted for use in other educational formats such as a longitudinal lecture series (as it was initially implemented) or as an accompaniment to a longitudinal clinic experience [21]. Finally, the majority of the curricular components are adaptable to the virtual environment and the core lectures are available as pre-recorded asynchronous videos; features desirable to site directors working to address the ACGME mandate for addiction medicine curricula in other IM residency programs [22].

Our pilot study results, however, have several limitations. While comparable to other survey-based evaluations of medical education interventions, our 39% response rate is low which, along with the voluntary nature of study participation, makes our results vulnerable to responder bias. Though our pre- post- design demonstrates a change following the rotation experience, our evaluation lacks comparison to a control group and is thus also susceptible to confounding bias. Additionally, interpretation of our results should be limited, as self-reported changes in perceptions and attitudes may not correspond to changes in actual knowledge or behavior. Finally, our findings represent the experience of a single institution and faculty educator, where a full week of the core residency calendar was dedicated to addiction medicine, and may not be replicable in other settings.

Our findings add to prior work utilizing stigma scales to explore the effect of educational interventions on attitude change toward people with SUDs, summarized in Table 3. In an early study evaluating the effect of a 10-hour addiction medicine didactic curriculum on internal medicine resident attitudes, a modest 0.16 point mean summative MCRS score increase for ‘alcoholism’ and no statistically significant change for ‘dependence on narcotic pain medication’ were observed; this curriculum, notably, did not have any stigma-specific learning objectives [13]. In contrast, an evaluation of medical students who received an anti-stigma curriculum from specialty faculty during a 3-week clerkship on an addiction psychiatry consult service found a significant 5.7 point mean summative MCRS score increase for ‘patients with substance use disorder’ [20]. Briefer anti-stigma educational interventions without clinical experiences have also been studied, with mixed findings: one study of an 8-minute online video training module about stigma and SUDs significantly increased IM and psychiatry resident MCRS scores from baseline to six-months for both opioid and alcohol use disorders [19], but another study of a 25-minute online interactive curriculum about stigmatizing language in clinical documentation did not shift IM resident attitudes towards people with SUDs on a 9-item stigma scale [23].

**Table 3:** Summary of associations between addiction medicine educational interventions and stigma-scale outcomes reported in the peer-reviewed literature

Study	Exposure	Pre-post Mean Summative Stigma Scale Outcome (Condition, mean overall sum/likert, *p*)
Meltzer 2013 [13]	10-hour addiction medicine didactic curriculum; no stigma-focused learning objectives or hands-on clinical experience	‘Dependence on narcotic pain medication’ MCRS sum: 35.3 to 35.4 (Δ = 0.09, *p* = 0.1) ‘Alcoholism’: MCRS sum: 41.4 to 41.56 (Δ = 0.16, *p* = 0.04)
Avery 2019 [19]	8-minute online video training module about stigma in SUDs; no clinical experience	Opioid use disorder MCRS Likert: 3.41 to 4.61 (Δ = 1.2, *p* < 0.001) Alcohol use disorder MCRS Likert: 3.74 to 4.86 (Δ = 1.12, *p* < 0.001)
Samberg 2025 [23]	25-minute online interactive module about stigma change in clinical documentation	‘People using drugs’ Attitude Measurement Total Score*: 15.40 to 15.23, Δ= − 0.16, *p* = 0.626
Lew 2024 [20]	Individual anti-stigma curriculum and 3-week addiction psychiatry consult clinical experience	‘Patients with SUD’ MCRS sum: 45.8 to 51.5 (Δ = 5.7, *p* = 0.0005)
Lawrence 2025 (Present study)	5-hours of video didactics, 3 h of facilitated workshops, within 1 week of clinical experience (2 days specialty SUD outpatient, 3 days inpatient addiction consult)	Opioid use disorder MCRS Likert: 3.81 to 4.29, (Δ = 0.48, *p* = 0.003 MCRS Sum: 41.94 to 47.17, (Δ = 5.23, *p* = 0.003 Alcohol use disorder MCRS Likert: 3.75 to 4.28, (Δ = 0.53, *p* < 0.001 MCRS Sum: 41.28 to 47.06, (Δ = 5.8, *p* < 0.001

The Attitude Measurement scale is reverse-scored, so higher values are associated with higher levels of stigma

Overall, these literature and our own findings suggest that both stigma-focused learning objectives and hands-on clinical experiences in addiction medicine settings—either outpatient or, increasingly, among inpatient addiction consult services—may be important ingredients for significant change in medical trainee attitudes towards people with SUDs.

While the results from our pilot study signal an important association between stigma-disrupting messaging strategies, addiction medicine clinical experience, and attitude change, further research is needed to better understand which components of this educational experience are most important, and whether their effects are sustained over time and associated with changes in knowledge and behavior. Our findings and prior research suggests, however, that if stigma-focused learning objectives are not incorporated into educational experiences, attitudes are unlikely to improve. Where appropriate, Internal Medicine Residency program and site directors may therefore consider incorporating stigma-disrupting learning objectives into their ACGME-mandated addiction medicine curricular experiences.

## Conclusions

In conclusion, this pilot study of a one-week IM resident core clinical rotation in addiction medicine incorporating a curriculum with stigma-disrupting learning objectives and messaging strategies was associated with significant increases in learner’s self-reported preparedness to diagnose and treat SUDs and positive regard towards patients with SUDs. Though further evaluation to identify optimal educational strategies are needed, IM educators developing ACGME-mandated addiction medicine curricula and seeking to improve resident confidence and attitudes towards patients with SUDs should consider including stigma-focused learning objectives and, where feasible, hands-on clinical experiences in addiction medicine.

## Supplementary Information

Supplementary Material 1.

## Data Availability

The datasets used and/or analysed during the current study are available from the corresponding author on reasonable request. Detailed materials for implementing the curriculum are also available from the corresponding author upon request.

## Abbreviations

ACGME: American College of Graduate Medical Education
AUD: Alcohol Use Disorder
IM: Internal Medicine
MCRS: Medical Condition Regard Scale
OUD: Opioid Use Disorder
SUD: Substance Use Disorder
