# Supplementary material for: Impact of a stigma-disrupting addiction medicine core curriculum on internal medicine resident attitudes and preparedness: results from a pilot study
Source: BMC Med Educ. 2026 Aug 12;26:1819. doi: 10.1186/s12909-026-10119-8 (PMC13750034; doi:10.1186/s12909-026-10119-8)
Supplement: Supplementary file 1 [file 12909_2026_10119_MOESM1_ESM.docx]

**Appendix A: Core Competencies in Unhealthy Substance Use for Internal Medicine Residents (Jackson, et al., 2010)**

1) Residents will perform age, gender and culturally appropriate unhealthy substance use screening

2) Residents will effectively assess patients with unhealthy substance use

3) Residents will provide brief interventions to patients with unhealthy substance use

4) Residents will demonstrate effective counseling methods to help prevent unhealthy substance use

5) Residents will refer patients with substance use disorders to treatment settings that provide pharmacotherapy for relapse prevention

6) Residents will recognize, treat or refer co-morbid medical and psychiatric conditions in patients with substance use conditions

7) Residents will refer patients with substance use disorders to appropriate treatment and supportive services

8) Residents will be aware of the ethical and legal issues around physician impairment from substance use and of resources for referring potential impaired colleagues, including employee assistance programs, hospital based committees, and state physician health programs and licensure boards

9) Residents will identify the legal and ethical issues involved in the care of patients with unhealthy substance use

10) Residents will provide pharmacologic withdrawal to patients with substance dependence

11) Residents will provide or refer for treatment for relapse prevention in patients with substance use disorders, both pharmacotherapy and psychosocial counseling

**Appendix B: Curriculum Learning Objectives (corresponding core competencies noted in parentheses)**

Asynchronous Video: Fundamentals of Substance Use Disorders Care (1, 6)

1. Describe the biopsychosocial model of addiction

2. Describe the impact of stigma on patients with substance use disorders

3. Use principles of trauma-informed care in management of patients with SUD

4. List harm reduction strategies for people who use substances

Facilitated Workshop: SBIRT and Core Motivational Interviewing Skills (1, 2, 3, 4, 5)

1. State the current prevalence of alcohol and drug use disorders

2. Pair-off and practice screening for and following a positive screen with a substance use history

3. Use the DSM-V criteria to diagnose a substance use disorder

4. Practice performing brief negotiated intervention

5. Simulate use of core motivational interviewing skills: elicit-provide-elicit, open-ended questions, affirmations, reflective statements, summarizing statements

Asynchronous Video: Policy Issues in Substance Use Disorders

1. Describe the current legal status of controlled substances

2. Identify the influence of systemic racism on the current legal status of and penalties for controlled substance use

3. Identify levels of care for SUD according to the ASAM continuum

4. Identify the ASAM criteria as a tool for determining medical necessity/level of care

5. Describe inequities in the use of urine drug testing

6. State some benefits and drawbacks of urine drug testing in patients with SUD

Facilitated Workshop: Ethical issues in Substance Use Disorder Care (8, 9)

1. Define the four ethical principles: (beneficence, nonmaleficence, autonomy, justice/fidelity)

2. Identify which ethical principles are in tension across different clinical scenarios, such as patients with: “drug-seeking” behaviors or a need for transplant or valve surgery due to complications of SUD

Asynchronous Video: Opioid Use Disorder: (5, 6, 7, 10, 11)

1. Identify and treat opioid withdrawal

2. Name the 3 FDA-approved medications for treatment of opioid use disorder (OUD) and their mechanisms of action

3. Compare and contrast buprenorphine and methadone for OUD

4. Name the two most common buprenorphine initiation strategies

5. Identify legal limitations on buprenorphine and methadone prescribing

6. List treatment resources for a patient with OUD

7. Manage acute pain in a patient with OUD

Asynchronous Video: Alcohol Use Disorder (5,6,7,10,11)

1. Identify candidates for ambulatory alcohol withdrawal management

2. Identify and treat alcohol withdrawal

3. Differentiate between alcoholic hallucinosis, delirium tremens, Wernicke encephalopathy, and Korsakoff syndrome

4. List treatment resources for a patient with AUD

5. List the 3 FDA-approved medications for treatment of alcohol use disorder and 2 off-label medication options

Asynchronous Video: Stimulant and Sedative Hypnotic Use Disorder (6, 7, 10, 11)

1. Describe current trends in misuse of sedative-hypnotics and stimulants

2. List medical complications of sedative-hypnotic use

3. Manage sedative-hypnotic use disorder in the inpatient and outpatient settings

4. List medical complications of stimulant use disorder

5. Manage stimulant withdrawal in the inpatient setting

6. Name 3 off-label medications and 3 behavioral treatments with evidence for treating stimulant use disorder

Facilitated Discussion: Reflections in Care of People with SUDs

1. Engage in reflective writing on a personal or clinical encounter with someone with SUD

2. Identify adjectives used to describe the person with SUD in the encounter described and imagine how the provider may have been perceived in turn^1^

**Appendix C: Pre- Post- Rotation Survey Instrument**

Section A

A1: What year are you in residency?

[] PGY 1

[] PGY 2

[] PGY 3

Section B

B1 (pre-survey only): Did your medical school offer any formal training in Addictions?

[] Yes

[] No

[] Don’t know

B2 (pre-survey only): During medical school, which of the following types of instruction did you receive in Addiction medicine?

(Please check all that apply)

[] A single lecture

[] A lecture series or dedicated learning block/elective

[] Clinical care for addicted populations

[] Online education or modules

[] Working in or shadowing at a facility dedicated to addictions treatment (i.e. methadone maintenance program)

[] Attending a recovery meeting

[] Other not listed here

[] My school did not offer any formal training or I don’t know

Section C

C8: Please note the extent to which you feel prepared to diagnose addiction:

[] Very prepared

[] Somewhat prepared

[] Somewhat unprepared

[] Very unprepared

C9: Please note the extent to which you feel prepared to treat addiction:

[] Very prepared

[] Somewhat prepared

[] Somewhat unprepared

[] Very unprepared

Section D

Use the scale below to rate your degree of agreement or disagreement with each of the following items regarding patients with **opioid use disorder:**

D1: Working with patients like this is satisfying.

Strongly Disagree [1]	[2]	[3]	[4]	[5]	[6] Strongly Agree

D2: Insurance plans should cover patients like this to the same degree that they cover patients with other conditions.

Strongly Disagree [1]	[2]	[3]	[4]	[5]	[6] Strongly Agree

D3: There is little I can do to help patients like this.

Strongly Disagree [1]	[2]	[3]	[4]	[5]	[6] Strongly Agree

D4: I feel especially compassionate toward patients like this.

Strongly Disagree [1]	[2]	[3]	[4]	[5]	[6] Strongly Agree

D5: Patients like this irritate me

Strongly Disagree [1]	[2]	[3]	[4]	[5]	[6] Strongly Agree

D6: I wouldn’t mind getting up on call nights to care for patients like this

Strongly Disagree [1]	[2]	[3]	[4]	[5]	[6] Strongly Agree

D7: Treating patients like this is a waste of medical dollars.

Strongly Disagree [1]	[2]	[3]	[4]	[5]	[6] Strongly Agree

D8: Patients like this are particularly difficult for me to work with.

Strongly Disagree [1]	[2]	[3]	[4]	[5]	[6] Strongly Agree

D9: I can usually find something that helps patients like this feel better.

Strongly Disagree [1]	[2]	[3]	[4]	[5]	[6] Strongly Agree

D10: I enjoy giving extra time to patients like this.

Strongly Disagree [1]	[2]	[3]	[4]	[5]	[6] Strongly Agree

D11: I prefer not to work with patients like this.

Strongly Disagree [1]	[2]	[3]	[4]	[5]	[6] Strongly Agree

Section E

Use the scale below to rate your degree of agreement or disagreement with each of the following items regarding patients with **alcohol use disorder:**

E1: Working with patients like this is satisfying.

Strongly Disagree [1]	[2]	[3]	[4]	[5]	[6] Strongly Agree

E2: Insurance plans should cover patients like this to the same degree that they cover patients with other conditions.

Strongly Disagree [1]	[2]	[3]	[4]	[5]	[6] Strongly Agree

E3: There is little I can do to help patients like this.

Strongly Disagree [1]	[2]	[3]	[4]	[5]	[6] Strongly Agree

E4: I feel especially compassionate toward patients like this.

Strongly Disagree [1]	[2]	[3]	[4]	[5]	[6] Strongly Agree

E5: Patients like this irritate me.

Strongly Disagree [1]	[2]	[3]	[4]	[5]	[6] Strongly Agree

E6: I wouldn’t mind getting up on call nights to care for patients like this

Strongly Disagree [1]	[2]	[3]	[4]	[5]	[6] Strongly Agree

E7: Treating patients like this is a waste of medical dollars.

Strongly Disagree [1]	[2]	[3]	[4]	[5]	[6] Strongly Agree

E8: Patients like this are particularly difficult for me to work with.

Strongly Disagree [1]	[2]	[3]	[4]	[5]	[6] Strongly Agree

E9: I can usually find something that helps patients like this feel better.

Strongly Disagree [1]	[2]	[3]	[4]	[5]	[6] Strongly Agree

E10: I enjoy giving extra time to patients like this.

Strongly Disagree [1]	[2]	[3]	[4]	[5]	[6] Strongly Agree

E11: I prefer not to work with patients like this.

Strongly Disagree [1]	[2]	[3]	[4]	[5]	[6] Strongly Agree
